# Baseline characteristics in the Israel refraction, environment, and devices (iREAD) study

**DOI:** 10.1038/s41598-023-29563-3

**Published:** 2023-02-17

**Authors:** Einat Shneor, Lisa A. Ostrin, Ravid Doron, Julia S. Benoit, Jonathan Levine, Kevin Davidson, Ariela Gordon-Shaag

**Affiliations:** 1 Department of Optometry, Hadassah Academic College, Haniviim St. 37, 9101001 Jerusalem, Israel; 2 College of Optometry, University of Houston, Houston, TX 77004 USA; 3 Texas Institute for Measurement, Evaluation, and Statistics, Houston, TX 77004 USA

**Keywords:** Epidemiology, Risk factors, Refractive errors, Paediatric research

## Abstract

The purpose of this study is to present baseline data from a longitudinal study assessing behavioral factors in three groups of boys in Israel with varying myopia prevalence. Ultra-Orthodox (N = 57), religious (N = 67), and secular (N = 44) Jewish boys (age 8.6 ± 1.4 years) underwent cycloplegic autorefraction and axial-length measurement. Time-outdoors and physical-activity were assessed objectively using an Actiwatch. Ocular history, educational factors, and near-work were assessed with a questionnaire. Group effects were tested and mixed effects logistic and linear regression were used to evaluate behaviors and their relationship to myopia. The prevalence of myopia (≤ − 0.50D) varied by group (ultra-Orthodox: 46%, religious: 25%, secular: 20%, P < 0.021). Refraction was more myopic in the ultra-Orthodox group (P = 0.001). Ultra-Orthodox boys learned to read at a younger age (P < 0.001), spent more hours in school (P < 0.001), spent less time using electronic devices (P < 0.001), and on weekdays, spent less time outdoors (P = 0.02). Increased hours in school (OR 1.70) and near-work (OR 1.22), increased the odds of myopia. Being ultra-Orthodox (P < 0.05) and increased near-work (P = 0.007) were associated with a more negative refraction. Several factors were associated with the prevalence and degree of myopia in young boys in Israel, including being ultra-Orthodox, learning to read at a younger age, and spending more hours in school.

## Introduction

Myopia is the most common cause of visual impairment worldwide^1,2^. The prevalence of myopia is increasing, particularly in urban populations, such as those in Eastern Asia, the United States, and, of interest to the current study, Israel^3^. Myopia is expected to affect 50% of the world population by 2050^1^. The prevalence of high myopia (≤ − 5.00D) is also increasing^4^, with a reported eightfold increase from the 1970s to the 2000s^5^, Myopia is associated with an increased risk of potentially blinding ocular pathologies, including glaucoma, myopic maculopathy, and posterior staphyloma, with the odds ratio of associated pathologies increasing with the degree of myopia^6^. With myopia tending to onset at younger ages in recent years, there is a greater risk of more individuals progressing to high myopia^7^.

Myopia is known to be due to a complex interaction between genetic and behavioral factors^8^. Children’s refractive error is strongly associated with the number of myopic parents^9^. However, the prevalence is increasing faster than genetics alone can account for, thereby implicating a role of environment and behavior^10^. Recent studies show that decreased time outdoors is associated with myopia onset^11,12^, with some studies also showing that increased time outdoors slows myopia progression^12,13^. Evidence for a role of near work is less clear, with some studies reporting associations between increased near work and screen time with myopia^14,15^, and others reporting no correlations^16–18^. Conflicting findings likely exist because children’s behaviors are difficult to precisely quantify due to the subjective nature in which data are typically measured.

It has previously been shown that the ultra-Orthodox population in Israel has a high prevalence of myopia^19^. A large population-based survey of 17-year-old Israeli Jewish boys (N = 22,823) demonstrated that ultra-Orthodox and religious boys have a higher prevalence of myopia (82.2 and 50.3%, respectively) than secular boys, with a prevalence of 29.7%, which is similar to the global average ^19^. This divergent rate of myopia in different populations of Israeli boys has been reported in several studies^19–22^ and is thought to be a result of the study habits of Jewish boys rather than genetic factors.

The Jewish population is genetically homogenous^23,24^ but has three distinct lifestyles and educational systems, each associated with a different prevalence of myopia^19,22^. The education system for Ultra-Orthodox boys involves intensive sustained near-work activity and long school days beginning at the age of three^25^. On the other hand, both boys and girls in the religious and secular systems, as well as ultra-Orthodox girls, only begin formal education at the age of six^26^. Their school system resembles a modern Western school system in terms of curriculum and time spent in school^27^, although religious schools have 2 to 3 weekly study hours more than secular schools, which is dedicated to intensive reading of religious texts. Religious schools are single-sex education while secular schools are co-ed^27^. In addition, ultra-Orthodox, religious, and secular Jewish groups have differing attitudes toward the use of electronic devices, which may also impact myopiagenic exposures.

In a pilot study, we previously reported that in a small group of ultra-Orthodox, religious, and secular Jewish boys in Israel, aged 8.5 to 12 years^21^, there were no statistically significant differences observed in physical activity or time spent outdoors between the groups. However, the groups demonstrated distinct educational demands, as expected, with findings suggesting that increased daily time at school and/or learning to read at an early age may contribute to previously reported differences in refractive error between groups. The pilot study had a small sample size and measured refraction without cycloplegia. Additionally, the pilot study only included children above the age of 8.5 years. A recent study found that outdoor light exposure is of particular importance to prevent myopia in 6–8-year-old children^28^. Thus, it is relevant to assess the differences between ultra-Orthodox, religious, and secular boys at a younger age and in a larger population.

In the current paper, we report the baseline characteristics and behaviors of boys enrolled in the Israel Refraction, Environment, and Devices (iREAD) Study. The iREAD Study aims to longitudinally study behavioral characteristics and their relationship to refractive error in three groups of young Israeli Jewish boys, ultra-Orthodox, religious, and secular. This will be achieved by studying clinical parameters, behaviors, and visual activity. This report presents three main findings: (1) baseline demographic and clinical characteristics of the children enrolled in the study, (2) comparisons of behaviors, including objective measures of light exposure, time outdoors, and physical activity and subjective reports of near work and electronic device use, and (3) modeling the relationships between refractive error, behaviors, and group.

## Results

### Baseline characteristics of participants

**Figure 1:**
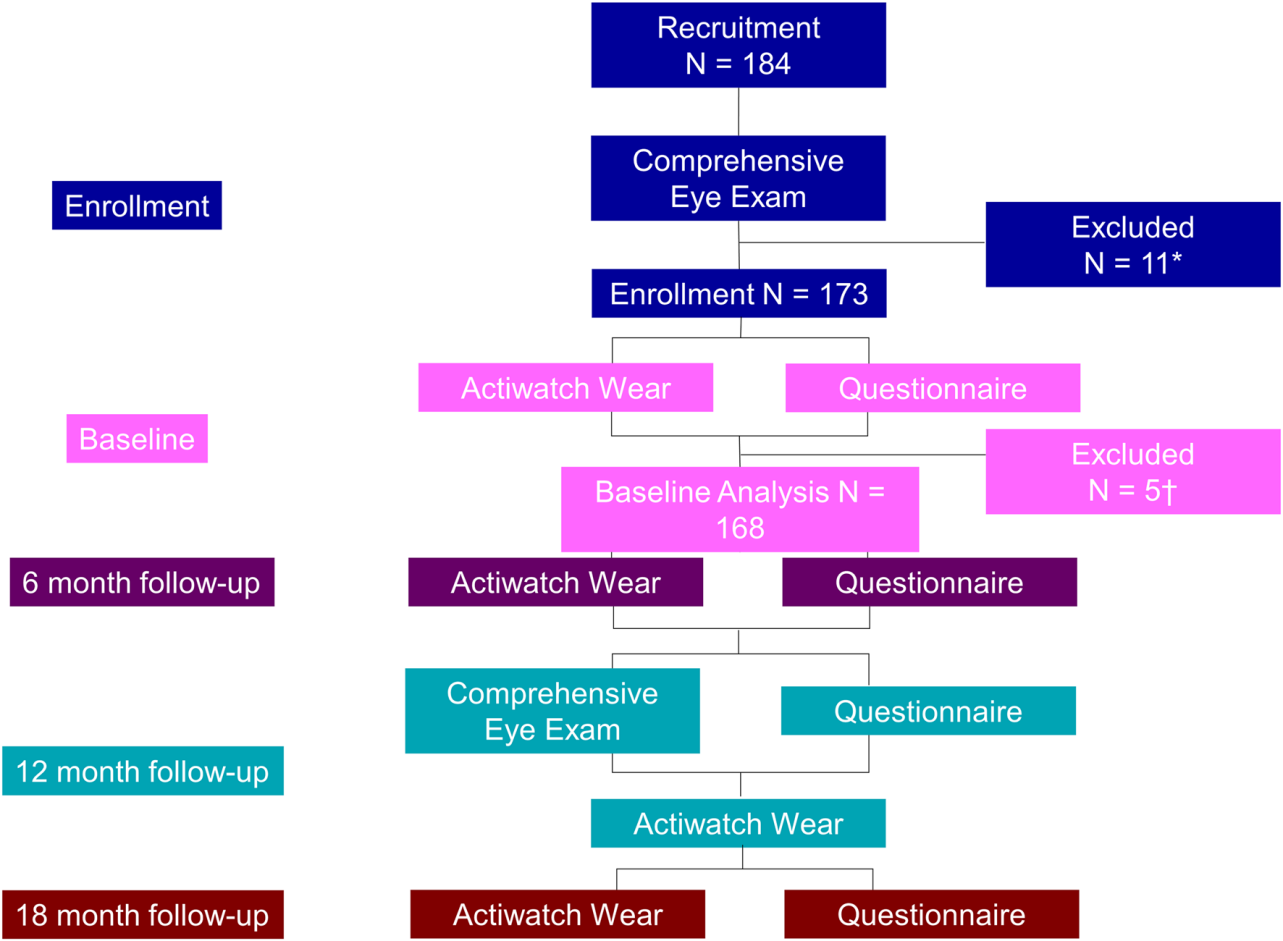
Protocol, recruitment, and enrollment for the 18-month iREAD study. Baseline analysis (N = 168) is presented in this paper. *11 boys were excluded: 3 had less than 6/9 acuity, 1 had less than 6/9 acuity and high astigmatism, 4 had high hyperopia, 3 had high astigmatism. ^†^5 boys were excluded: 4 provided less than 4 days of valid Actiwatch data and 1 did not fill out the questionnaire.

The protocol, recruitment, and enrollment are shown in Fig. 1. A total of 184 boys were recruited to the study (ultra-Orthodox: N = 61, religious: N = 73, and secular: N = 50), among which 173 (94%) met inclusion criteria and were enrolled. Eleven boys were excluded for not meeting the inclusion criteria despite passing the pre-screening recruitment questionnaire to exclude hyperopia and amblyopia; 3 ultra-Orthodox boys had less than 6/9 best corrected visual acuity, 1 secular boy had less than 6/9 best corrected visual acuity and high astigmatism, 4 religious boys had hyperopia higher than + 2.50 D spherical equivalent, and 3 secular boys had high astigmatism. In addition, 5 boys were excluded from the study for technical reasons: the parents of 1 secular boy did not fill out the questionnaire, and 4 boys (1 ultra-Orthodox, 2 religious, and 1 secular) had less than 4 days of valid Actiwatch data. Thus, the final sample included 168 boys (ultra-Orthodox: N = 57, religious: N = 67, and secular: N = 44) from 133 families. Characteristics of the participants are presented in Table 1. There was no statistically significant difference in age between groups (P = 0.428). With regards to time of year of participation in baseline measures, when analyzing wear time at the  family and individual child, there were no statistical differences across groups for temperature (child: P = 0.496; family: P = 0.493), number of daylight hours (child: P = 0.589; family: P = 0.583), or rainfall (child: P = 0.502; family: P = 0.770).

**Table 1:** Demographic and clinical information (mean ± SD and range) overall and by religious group for the boys from the iREAD study sample (N = 168).

	Total (N = 168)	Ultra-Orthodox (N = 57)	Religious (N = 67)	Secular (N = 44)	P value
Number of families	133	40	54	39	
Age (years)	8.6 ± 1.4 (5.1–12.0)	8.5 ± 1.4 (5.1–11.5)	8.7 ± 1.5 (6.0–12.0)	8.4 ± 1.3 (5.2–11.0)	P = 0.429
Myopes: non-myopes	52:116	26:31	17:50	9:35	P = 0.021 Post-hoc: UO > S, P = 0.037
Spherical equivalent refraction (D)	− 0.17 ± 1.42 (− 5.23 to + 2.38)	− 0.79 ± 1.72 (− 5.23 to + 2.38)	+ 0.08 ± 1.17 (− 2.96 to + 2.25)	+ 0.26 ± 1.04 (− 2.99 to + 2.35)	P < 0.001 Post-hoc: UO < R, P = 0.003; UO < S, P = 0.001
Axial length (mm)	23.35 ± 0.98 (21.11–26.13)	23.6 ± 1.07 (21.19–25.61)	23.27 ± 0.99 (21.11–26.13)	23.14 ± 0.77 (21.54–25.59)	P = 0.051
Day length (hours)	11.9 ± 1.5 (10.1–14.2)	11.9 ± 1.5 (10.1–14.2)	11.8 ± 1.4 (10.1–14.2)	12.1 ± 1.6 (10.1–14.2)	P = 0.589
Temperature (°C)	19.2 ± 6.5 (8.8–30.2)	18.8 ± 6.8 (9.1–29.8)	18.5 ± 6.4 (8.8–29.8)	20.5 ± 6.1 (9.2–30.2)	P = 0.496
Rainfall (mm per day)	1.5 ± 1.7 (0–7.5)	1.6 ± 1.8 (0–5.9)	1.6 ± 1.8 (0–7.5)	1.1 ± 1.4 (0–5.9)	P = 0.502

Spherical equivalent refraction and axial length from the right and left eyes were highly correlated (axial length: *r* = 0.978, P < 0.001; SE: *r* = 0.920, P < 0.001), and there were no statistically significant differences between the right and left eyes for axial length (P = 0.408) or spherical equivalent refraction (P = 0.282). Therefore, both eyes of each child were averaged and used in subsequent analyses^29^.

The prevalence of myopia for the entire population was 31.0% (ultra-Orthodox: 45.6%, religious: 25.3%, and secular: 20.4%, P = 0.021). The ultra-Orthodox group had a higher prevalence of myopia than the secular group (P = 0.037). No significant difference in prevalence was observed between the other groups. The spherical equivalent refraction of the ultra-Orthodox group (− 0.79D ± 1.72D) was significantly more myopic than the religious (+ 0.08D ± 1.17D, P = 0.003) and secular (+ 0.26D ± 1.04D, P = 0.001) groups (Table 1). While the axial length tended to be longer for ultra-Orthodox children, no statistical difference was observed across groups (P = 0.051).

### Educational and near work characteristics

The results of the UH NEAR questionnaire revealed differing backgrounds, educational characteristics, and behaviors between the ultra-Orthodox, religious, and secular groups (Table 2). Ultra-Orthodox boys were more likely to have myopic parents than the religious (P = 0.001) and secular boys (P < 0.001). Ultra-Orthodox boys learned to read at a significantly younger age than religious and secular boys (ultra-Orthodox: 4.4 ± 0.8 years; religious: 5.9 ± 0.6 years; secular: 6.1 ± 0.3 years; P < 0.001), and ultra-Orthodox boys spent more hours in school from Sunday to Friday. Additionally, ultra-Orthodox boys were less likely than secular boys to have a cellphone (P = 0.005).

**Table 2:** Responses from the UH NEAR questionnaire among boys from the iREAD study (n = 168), presented overall and by religious groups: ultra-Orthodox (N = 57), religious (N = 67), and secular (N = 44).

	Total (N = 168)		Ultra-Orthodox (N = 57)	Religious (N = 67)	Secular (N = 44)	P value
Parental myopia, N (%)						
0 myopic parents	25 (15.2)		0 (0.0)	14 (21.2)	11 (26.2)	P < 0.001*^†^ Post-hoc: UO > R, P = 0.001; UO > S, P < 0.001
1 myopic parents	57 (34.5)		14 (24.6)	24 (36.4)	19 (45.2)	
2 myopic parents	83 (50.3)		43 (75.4)	28 (42.4)	12 (28.6)	
Children with cell phones, N (%)	34 (20.2)		3 (5.3)	14 (20.9)	17 (38.7)	P = 0.006 Post-hoc: UO < S, P = 0.005
Age learned to read (years) mean ± SD, range	5.4 ± 1.0		4.4 ± 0.8	5.9 ± 0.6	6.1 ± 0.3	P < 0.001* Post-hoc: UO < R, P < 0.001; UO < S, P < 0.001
	3–7		3–6	4–7	5–7	
Following Parent-Reported Behaviors: Mean ± SD, Median (25th percentile, 75th percentile), range						
Time in school per day (hours)						
Overall	6.4 ± 1.0		6.8 ± 1.0	6.3 ± 0.8	6.0 ± 1.0	P < 0.001 Post-hoc: UO > S, P < 0.001; UO > R, P = 0.012
	6.5 (5.7, 7.3)		6.9 (6.4, 7.3)	6.2 (5.7, 7.1)	5.7 (5.7, 6.5)	
	3.3–8.3		3.8–8.2	4.5–8.3	3.3–8.2	
Weekday	6.9 ± 1.2		7.4 ± 1.2	6.8 ± 1.0	6.4 ± 1.2	< 0.001 Post-hoc: UO > S, P < 0.001; UO > R, P = 0.001
	7 (6, 8)		7.5 (7, 8)	6.8 (6, 8)	6 (6, 7)	
	4–9		4–9	5–9	4–9	
Friday	3.8 ± 0.8		3.7 ± 0.6	3.8 ± 1.0	3.9 ± 0.9	P = 0.480
	4 (3.5, 4)		3.5 (3, 4)	4 (4, 4)	4 (4, 4)	
	0–6		3–5	0–6	0–5.5	
Hand-held electronic device use (hours per day)						
Overall	0.8 ± 1.1		0.4 ± 0.7	0.8 ± 1.0	1.5 ± 1.4	P < 0.001 Post-hoc: UO < S, P < 0.001; R < S, P = 0.002
	0.9 (0, 1)		0 (0, 0.9)	0.9 (0, 0.9)	1.1 (0.9, 2)	
	0–6.1		0–4.7	0–3.4	0–6.1	
Weekday	0.9 ± 1.2		0.4 ± 0.8	0.9 ± 1.2	1.5 ± 1.5	P < 0.001 Post-hoc: UO < R, P = 0.033; UO < S, P < 0.001; R < S, P = 0.038
	1 (0, 1)		0 (0, 1)	1 (0, 1)	1 (1, 2)	
	0–7		0–5	0–4	0–7	
Shabbat	0.5 ± 1.1		± 0.4	0.0 ± 0.2	1.6 ± 1.6	P < 0.001 Post-hoc: UO < S, P < 0.001; R < S, P < 0.001
	0 (0, 0)		0 (0, 0)	0 (0, 0)	1 (0, 2)	
	0–7		0–3	0–2	0–7	
All screen use (hand-held devices, computer, TV/Video, hours per day)						
Overall	2.6 ± 3.2		0.9 ± 2.0	2.7 ± 2.6	4.6 ± 4.0	P < 0.001 Post-hoc: UO < S, P < 0.001; R < S, P = 0.002
	1.7 (0.9, 3.4)		0.9 (0, 0.9)	1.7 (.9, 4.3)	3.4 (2.4, 5.6)	
	0–18.4		0–14.1	0–10.3	0–18.4	
Weekday	2.8 ± 3.4		1 ± 2.1	3.1 ± 3.0	4.6 ± 4.20	P < 0.001 Post-hoc: UO < R, P < 0.001; UO < S, P < 0.001; R < S, P = 0.045
	2 (1, 3)		1, (0, 1)	2 (1, 5)	3 (2.5, 5.5)	
	0–21		0–15	0–12	0–21	
Shabbat	1.4 ± 3.2		0.3 ± 1.3	0.1 ± 0.4	5.0 ± 4.5	P < 0.001 Post-hoc: UO < S, P < 0.001; R < S, P < 0.001
	0 (0, 1)		0 (0, 0)	0 (0, 0)	4 (2, 6)	
	0–21		0–9	0–2	0–21	
Writing and reading printed material (hours per day)						
Overall	2.6 ± 2.1		2.9 ± 2.3	2.4 ± 2.0	2.4 ± 1.9	P = 0.429
	2 (1.1, 3)		2.1 (1.1, 3)	2 (1, 2.9)	2 (1.1, 3.1)	
	0–12		0.1–12	0–11.7	0–10.1	
Weekday	2.7 ± 2.3		3.0 ± 2.5	2.5 ± 2.2	2.4 ± 1.9	P = 0.380
	2 (1, 3)		2 (1, 3)	2 (1, 3)	2 (1, 3)	
	0–13		0–12	0–13	0–10	
Shabbat	2.0 ± 1.8		2.0 ± 1.7	1.7 ± 1.3	2.3 ± 2.4	P = 0.287
	2 (1, 2)		2 (1, 3)	1 (1, 2)	2 (1, 2)	
	0–12		0–12	0–7	0–11	
All near work, printed and electronic < 40 cm (reading, writing, hand-held electronic devices, hours per day)						
Overall	3.4 ± 2.6		3.2 ± 2.7	3.2 ± 2.2	3.9 ± 3.0	P = 0.429
	2.9 (1.9, 3.9)		2.9 (1.9, 3.7)	2.7 (1.9, 3.9)	3 (2, 4.5)	
	0.1–16.7		0.1–16.7	0.9–11.7	1–16.3	
Weekday	3.6 ± 2.8		3.4 ± 2.9	3.4 ± 2.5	3.9 ± 3.0	P = 0.696
	3 (2, 4)		3 (2, 4)	3 (2, 4)	3 (2, 5)	
	0–17		0–17	1–13	1–16	
Shabbat	2.4 ± 2.5		2.1 ± 2.0	1.7 ± 1.3	3.9 ± 3.7	P < 0.001 Post-hoc: S > R, P < 0.001; S > UO, P < 0.001
	2 (1, 3)		2 (1, 3)	1 (1, 2)	3 (2, 4)	
	0–18		0–15	0–7	0–18	
Intermediate near work 40-100 cm (card and board games, computer use, hours per day)						
Overall	2.1 ± 1.7		1.6 ± 1.1	2.3 ± 1.5	2.7 ± 2.2	P = 0.006 Post-hoc: S > UO, P = 0.004
	2 (1.1, 2.2)		1.3 (1, 2)	2 (1.1, 2.7)	2.1 (1.1, 3.1)	
	0–11.3		0.1–6.3	0.1–7.9	0–11.3	
Weekday	2.1 ± 1.7		1.6 ± 1.2	2.3 ± 1.7	2.6 ± 2.3	P = 0.01 Post-hoc: R > UO, P = 0.04; S > UO, P = 0.01
	2 (1, 2)		1 (1, 2)	2 (1, 3)	2 (1, 3)	
	0–11		0–6	0–8	0–11	
Shabbat	2.2 ± 1.7		1.9 ± 1.3	1.9 ± 1.2	3.2 ± 2.3	P < 0.001 Post-hoc: S > UO, P < 0.001; S > R, P < 0.001
	2 (1, 3)		2 (1, 2)	2 (1, 2)	3 (2, 3.5)	
	0–13		0–8	0–7	0–13	
Far viewing (television and video games) > 100 cm (hours per day)						
Overall	0.9 ± 1.2		0.2 ± 0.8	0.8 ± 1.1	1.8 ± 1.3	P < 0.001 Post-hoc: S > R, P < 0.001; S > UO, P < 0.001; R > UO, P = 0.004
	0 (0, 10.1)		0 (0, 0)	0 (0, 1.7)	1.1 (1, 2)	
	0–6.1		0–5.4	0–5.1	0–6.1	
Weekday	0.9 ± 1.3		0.2 ± 0.9	1.0 ± 1.3	1.7 ± 1.4	P < 0.001 Post-hoc: S > R, P = 0.002; S > UO, P < 0.001; R > UO, P = 0.002
	0 (0, 1)		0 (0, 0)	0 (0, 2)	1 (1, 2)	
	0–7		0–6	0–6	0–7	
Shabbat	0.6 ± 1.2		0.1 ± 0.3	0 ± 0	2.0 ± 1.6	P < 0.001 Post-hoc: S > R, P < 0.001; S > UO, P < 0.001
	0 (0, 0)		0 (0, 0)	0 (0, 0)	2 (1, 2.5)	
	0–7		0–2	0–0	0–7	

Weekdays included Sunday through Friday afternoon, when children were typically in school, and Shabbat included Friday evening through Saturday, when children were out of school. Shabbat begins about one hour before sunset on Friday and ends at sunset on Saturday.

*SD* standard deviation.

^†^For three families the data were not available due to adoption.

**Figure 2:**
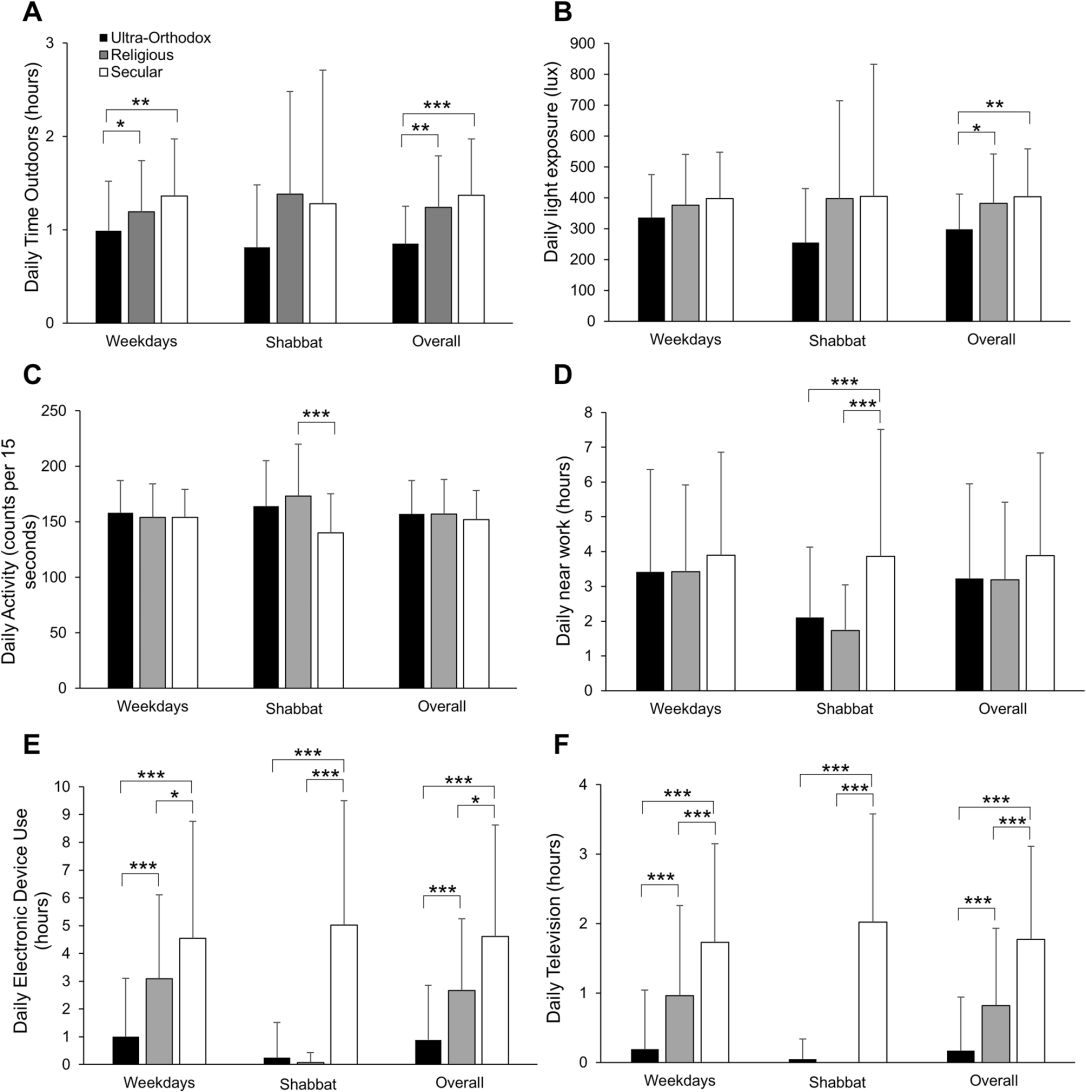
Environmental and behavioral measures. Mean ± standard deviation daily (**A**) time outdoors (hours), (**B**) light exposure (lux), (**C**) activity (counts per 15 s), (**D**) near work (hours), (**E**) electronic device use (hours), and (**F**) television (hours), are shown for weekdays, Shabbat, and the whole week (overall) for ultra-Orthodox (filled bars), religious (gray bars), and secular (open bars) children; P < 0.05, **P < 0.01, ***P < 0.001 for Bonferroni-corrected post hoc pairwise comparisons.

There were no differences between groups for parent reported time spent writing and reading outside of school for weekdays, Shabbat, or overall, nor were there differences in all near work (reading, writing, and near devices) for weekdays or overall (P > 0.05 for all, Table 2 and Fig. 2). However, secular boys engaged in significantly more near work on Shabbat than ultra-Orthodox and religious boys (ultra-Orthodox: 2.1 ± 2.0 h; religious: 1.7 ± 1.1 h; secular: 3.7 ± 3.7 h, P < 0.001). There was a significant difference between all groups for the use of hand-held devices on the weekdays and overall. Overall, ultra-Orthodox boys spent less time using devices than religious and secular boys, and religious boys spent less time using devices than secular boys (ultra-Orthodox: 0.4 ± 0.7 h; religious: 0.8 ± 1.0 h; secular: 1.5 ± 1.4 h; P < 0.02). On Shabbat, there were no differences in the use of hand-held devices between ultra-Orthodox and religious boys, but secular children spent significantly more time on devices than both ultra-Orthodox and religious boys (ultra-Orthodox: 0.1 ± 0.4 h; religious: 0.03 ± 0.2 h; secular 1.6 ± 1.6 h; P < 0.001). A similar pattern was observed for all screen use, which included hand-held devices, computers, and televisions.

Ultra-Orthodox boys spent significantly less time overall than secular or religious boys engaged in intermediate near work from 40 to 100 cm, which included card games, board games, and computer use (P < 0.001). Divergent behavior between the three groups emerged for overall far viewing (> 100 cm, TV/video games, P < 0.001). In contrast, on Shabbat, ultra-Orthodox and religious boys had similar intermediate and far work, which was significantly less than the secular boys.

### Light exposure and physical activity characteristics

Objective behavioral data derived from the Actiwatch are shown in Table 3. On average, children provided 9.8 ± 2.4 days and 11.5 ± 2.3 nights of valid Actiwatch data. No statistically significant differences were observed between the groups for number of days (P = 0.16) or nights (P = 0.12). However, for religious reasons, some ultra-Orthodox (N = 23) and religious (N = 4) boys did not wear the Actiwatch on Shabbat. Furthermore, the data from one secular child was not valid for Shabbat. Thus, observations for only 140 children are included for analyses of Actiwatch data for Shabbat and overall (weekday + Shabbat) during the day and 142 children for sleep parameters.

**Table 3:** Objective measurements (mean ± SD and range) from the Actiwatch among boys from the iREAD study (N = 168), Shabbat (N = 140), and overall (N = 140).

	All (N = 168)	Ultra-Orthodox (N = 57)	Religious (N = 67)	Secular (N = 44)	P value
Valid days					
Overall	9.8 ± 2.4	9.2 ± 2.2	10.1 ± 2.4	10.2 ± 2.4	P = 0.163
	4–13	6–13	4–13	5–13	
Weekdays	8.5 ± 1.9	8.2 ± 1.7	8.6 ± 2.1	8.6 ± 2.1	n/a
	3–11	5–11	3–11	4–11	
Shabbat	1.4 ± 0.8	1.0 ± 0.9	1.5 ± 0.6	1.64 ± 0.5	n/a
	0–2	0–2	0–2	0–2	
Valid nights					
Overall	11.5 ± 2.3	10.8 ± 2.2	11.7 ± 2.4	12.0 ± 2.2	P = 0.117
	5–14	7–14	5–14	6–14	
Weeknights	9.9 ± 1.9	9.3 ± 1.9	10.1 ± 1.9	10.3 ± 1.8	n/a
	4–12	6–12	4–12	6–12	
Shabbat	1.6 ± 0.6	1.5 ± 0.5	1.6 ± 0.6	1.7 ± 0.5	n/a
	0–2	0–2	0–2	0–2	
Wake time					
Overall	06:52:04 ± 00:35:18	06:57:22 ± 00:33:30	06:50:08 ± 00:41:14	06:50:43 ± 00:26:28	P = 0.814
Weekdays	06:49:37 ± 00:33:09	06:58:36 ± 00:29:46	06:45:06 ± 00:37:33	06:44:51 ± 00:28:05	P = 0.135
Shabbat	07:34:22 ± 01:09:44	07:29:38 ± 01:01:57	07:33:29 ± 01:15:35	07:39:24 ± 01:07:49	P = 0.862
Bed time^£^					
Overall	21:23:38 ± 0:48:29	21:26:53 ± 0:40:51	21:20:02 ± 0:58:25	21:26:53 ± 0:40:51	P = 0.814
Weekdays	21:19:36 ± 0:46:29	21:27:44 ± 0:35:55	21:13:38 ± 0:58:02	21:18:08 ± 0:37:22	P = 0.280
Shabbat	22:11:24 ± 1:17:25	22:18:45 ± 1:20:34	22:00:44 ± 1:17:03	22:21:44 ± 1:15:09	P = 0.407
Sleep duration (hours)^£^					
Overall	9.1 ± 0.5	9.2 ± 0.5	9.1 ± 0.5	9.0 ± 0.5	P = 0.365
	7.6–10.3	7.9–10.3	7.6–10.0	7.6–10.0	
Weekdays	9.1 ± 0.6	9.2 ± 0.5	9.1 ± 0.6	9.0 ± 0.6	P = 0.648
	7.2–10.3	8.0–10.3	7.2–10.3	7.6–10.1	
Shabbat	8.9 ± 1.1	8.7 ± 1.0	9.2 ± 1.1	8.7 ± 1.0	P = 0.097
	5.4–11.3	5.6–10.7	5.4–11.3	5.9–10.7	
Daily average white light exposure (lux)					
Overall	368 ± 153	298 ± 114	382 ± 160	403 ± 156	P = 0.033 Post-hoc: UO < R, P = 0.037; UO < S, P = 0.011
	65–887	65–581	100–887	184–785	
Weekdays	368 ± 153	336 ± 139	376 ± 164	398 ± 150	P = 0.247
	59–832	59–699	95–832	166–738	
Shabbat	365 ± 334	255 ± 175	397 ± 317	405 ± 428	P = 0.231
	15–2152	43–747	15–1418	28–2152	
Time outdoors (hours)					
Overall	1.19 ± 0.57	0.85 ± 0.4	1.24 ± 0.55	1.37 ± 0.6	P = 0.002 Post-hoc: UO < R, P = 0.002; UO < S, P < 0.001
	0.12–2.88	0.12–1.8	0.17–2.88	0.25–2.78	
Weekdays	1.17 ± 0.57	0.99 ± 0.53	1.19 ± 0.55	1.36 ± 0.61	P = 0.018 Post-hoc: UO < R, P = 0.050; UO < S, P = 0.003
	0.09–2.79	0.09–2.21	0.16–2.54	0.26–2.79	
Shabbat	1.21 ± 1.14	0.81 ± 0.67	1.38 ± 1.1	1.28 ± 1.43	P = 0.097
	0–6.81	0.03–2.64	0.01–4.89	0–6.81	
Physical activity (counts per 15 s)					
Overall	156 ± 29	157 ± 30	157 ± 31	152 ± 26	P = 0.814
	85–229	85–207	94–229	107–207	
Weekdays	156 ± 28	158 ± 29	154 ± 30	154 ± 25	P = 0.787
	85–228	85–228	97–224	111–215	
Shabbat	161 ± 44	164 ± 41	173 ± 47	140 ± 35	P = 0.004 Post-hoc: S < R, P < 0.001
	81–363	84–245	81–364	83–224	
Moderate and vigorous activity (minutes per day)					
Overall	154 ± 47	153 ± 50	156 ± 49	149 ± 44	P = 0.835
	50–275	50–249	67–275	60–235	
Weekdays	153 ± 46	154 ± 48	152 ± 47	153 ± 43	P = 0.985
	53–268	53–256	71–268	63,246	
Shabbat	161 ± 69	166 ± 70	179 ± 71	129 ± 55	P = 0.004 Post = hoc: S < R; P < 0.001; S < UO; P = 0.046
	34–380	35–296	46–380	34–252	

^**£**^Data was available for 142 children for Shabbat nights.

Ultra-Orthodox boys spent significantly less time outdoors than religious and secular boys during the weekdays (ultra-Orthodox: 0.99 ± 0.53 h; religious: 1.19 ± 0.55 h; secular 1.36 ± 0.61 h, P = 0.02) and overall (ultra-Orthodox: 0.85 ± 0.4 h; religious: 1.24 ± 0.55 h; secular: 1.37 ± 0.6 h, P = 0.002), but with no differences on Shabbat. Furthermore, the ultra-Orthodox boys were exposed to significantly less average white light than secular boys (P = 0.01) and religious boys (P = 0.04) overall (ultra-Orthodox: 298 ± 114 lx; religious: 382 ± 160 lx; secular: 403 ± 156 lx), but not on weekdays or Shabbat. No statistically significant differences in physical activity (average CP15 and minutes of moderate and vigorous physical activity) emerged for weekdays or overall. However, secular boys were significantly less active than religious and ultra-Orthodox boys on Shabbat (ultra-Orthodox: 164 ± 41 CP15; religious: 173 ± 47 CP15; secular: 140 ± 35 CP15; P < 0.005). Secular boys had significantly fewer minutes of moderate and vigorous physical activity than religious boys (religious: 179 ± 71 min; secular: 129 ± 55 min, P < 0.001). For sleep measurements (bedtime, waketime, and sleep duration), there were no statistically significant differences between the groups for weekday, Shabbat, or overall.

To determine if younger children and older children had different behaviors, a secondary analysis was performed with age classified as younger (6–8 years) or older (9–10 years). There were no differential effects of group by age for Actiwatch metrics (P > 0.05 for all, data not shown).

**Figure 3:**
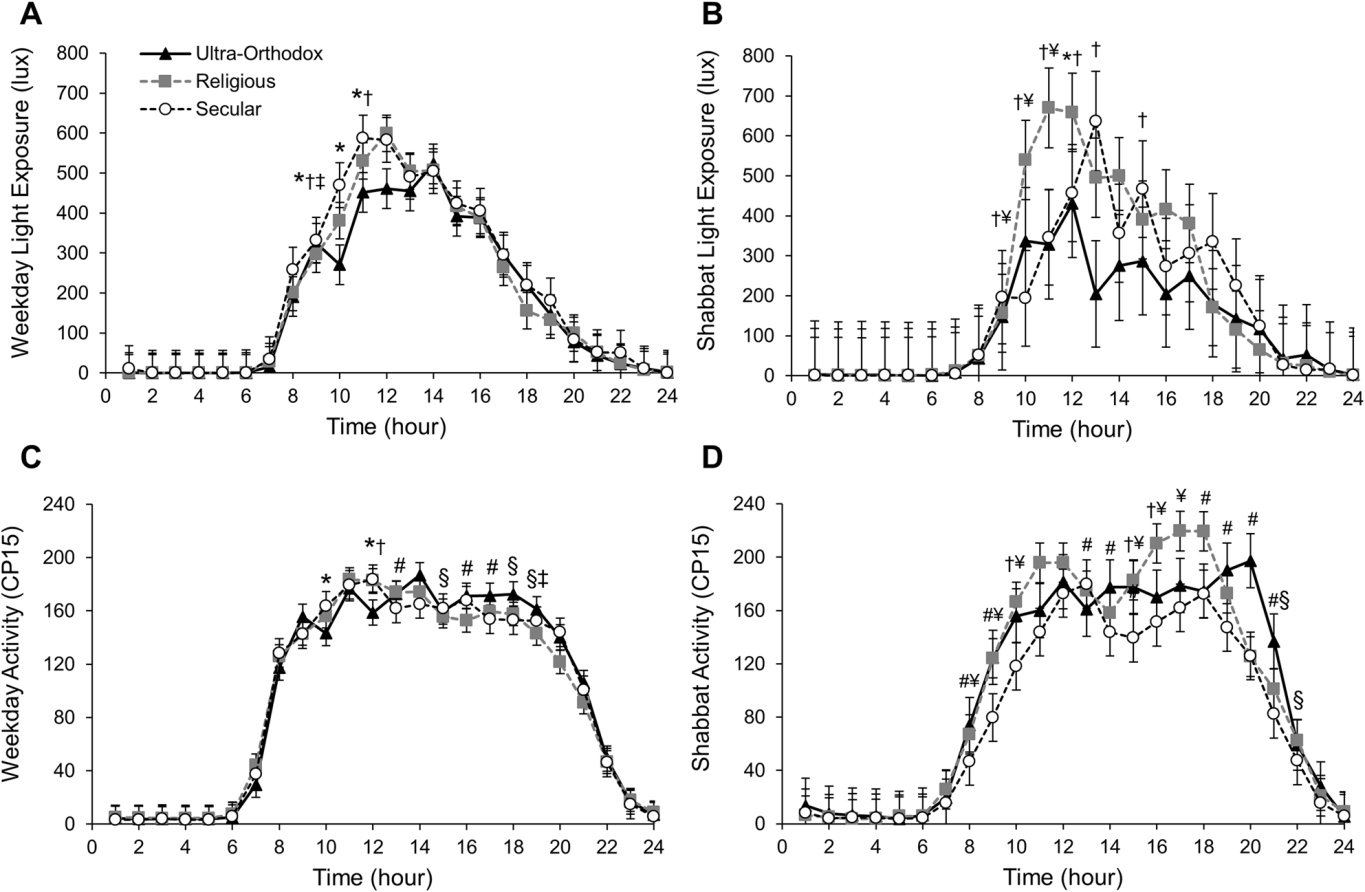
Activity in counts per 15 s (CP15) and light exposure (lux) analyzed by hour. Activity in counts per 15 s (CP15) and light exposure (lux) analyzed by hour for weekdays (**A,C**) and Shabbat (**B,D**) for ultra-Orthodox (UO, triangles), religious (R, squares), and secular (S, circles) groups; error bars represent 95% confidence intervals; post-hoc pairwise comparisons P < 0.05: *UO < S, ^†^UO < R, ^‡^R < S, ^¥^S < R, ^#^S < UO, ^§^R < UO.

Overall, there was a statistically significant differential effect of group on hourly differences of light exposure and activity on both weekdays and Shabbat (Fig. 3) tested via an hour by group interaction for each measure, separately. During the weekdays, ultra-Orthodox boys had less light exposure than secular and religious boys during the 9:00 and 11:00 h (P < 0.05 for all), while during the 10:00 h they had less light exposure than secular boys (P < 0.001). Religious boys had less light exposure than secular boys during the 9:00 h (P = 0.043). On Shabbat, ultra-Orthodox boys had less light exposure than religious boys during the 9:00 to 13:00 and 15:00 h (P < 0.05 for all); secular boys also had less light exposure during the 9:00 to 11:00 h than religious boys (P < 0.05 for all). Finally, during the 12:00 h, ultra-Orthodox boys had less light exposure than secular boys (P < 0.001).

During the weekdays, ultra-Orthodox boys had lower activity during the 9:00 and 11:00 h relative to secular boys, and the 11:00 h relative to religious boys (P < 0.05 for all). Secular boys had less activity than ultra-Orthodox boys during the 13:00, 16:00, and 17:00 h, and more than religious boys during the 15:00 and 18:00 h (P < 0.05 for all). Religious children had less activity than both ultra-Orthodox and secular boys during the 19:00 h (P < 0.05 for all). On Shabbat, secular boys had less activity during various morning and afternoon to evening hours relative to religious (8:00 to 10:00, 15:00 to 17:00) and ultra-Orthodox (8:00, 9:00, 13:00, 14:00, and 18:00 to 21:00) boys. Relative to ultra-Orthodox boys, religious boys had more activity during the 10:00, 15:00, and 16:00 h and less activity during the 21:00 and 22:00 h (P < 0.05).

### Relationships between group and behaviors with myopia

To understand the importance of group (ultra-Orthodox, religious, and secular) on myopia and the separate contributions of known risk factors (time outdoors, near work, age learned to read, and time in school) individual (univariable) and collective (multivariable) associations of group and behavioral characteristics on log odds of presence of myopia (myopic versus non-myopic) and spherical equivalent refraction, were evaluated as reported below.

### Presence of myopia

Univariable analyses showed that group was associated with odds of having myopia (Table 4); being secular or religious decreased the odds of having myopia compared to being ultra-Orthodox (religious versus ultra-Orthodox: (OR (95% CI)): 0.41 (0.19, 0.89); secular versus ultra-Orthodox: 0.31 (0.12, 0.78). In the presence of other behavioral characteristics in multivariable analyses, this finding was no longer statistically significant. Univariable analyses showed that myopia was associated with increased reported daily time in school (< 7 h versus > 7 h, OR 1.69 (1.22, 2.35)) and reading at an early age (< 6 years versus > 6 years, OR 2.22 (1.11, 4.42)). In multivariable analyses, which included behavioral characteristics and religious group, increased time in school was associated with higher odds of myopia. In the fully adjusted model (adjusted for age and parental myopia), daily near work (< 3.75 h versus > 3.75 h, OR 1.22 (1.01, 1.47)) and time in school (OR 1.70 (1.08, 2.67)) increased the odds of myopia. No statistically significant associations between group, time outdoors, or age at which the child learned to read were found in adjusted analyses.

**Table 4:** Associations of presence of myopia with exposure and behavioral characteristics of religious group, time outdoors, near work, age learned to read, and hours spent in school (N = 168).

Exposure and behavioral characteristics	Univariable analysis OR (95% CI)	P value	^a^Multivariable analysis OR (95% CI)	P value	^b^Adjusted Analysis OR (95% CI)	P value
Group		P = 0.020		P = 0.324		P = 0.549
Religious	0.41 (0.19, 0.89)	P = 0.025	0.39 (0.1, 1.49)	P = 0.162	0.55 (0.11, 2.67)	P = 0.446
Secular	0.31 (0.12, 0.78)	P = 0.014	0.30 (0.06, 1.64)	P = 0.159	0.68 (0.1, 4.48)	P = 0.678
Time outdoors (hours)	0.64 (0.34, 1.18)	P = 0.145	0.76 (0.38, 1.52)	P = 0.419	0.68 (0.3, 1.55)	P = 0.348
All near work (hours)	1.12 (0.99, 1.28)	P = 0.073	1.16 (1, 1.34)	P = 0.055	1.22 (1.01, 1.47)	P = 0.043
Early readers (< 6 years old)	2.22 (1.11, 4.42)	P = 0.025	0.66 (0.17, 2.57)	P = 0.542	0.58 (0.13, 2.68)	P = 0.472
Time in school (hours)	1.69 (1.22, 2.35)	P = 0.003	1.56 (1.09, 2.25)	P = 0.018	1.70 (1.08, 2.67)	P = 0.023

Reference categories: Religious group: ultra-Orthodox; Older Readers (> 6 years).

Each model was implemented with numerical and categorical exposure forms included into the model to safeguard model assumptions (not presented).

*OR* odds ratios, *CI* confidence intervals.

^a^Multivariable Analysis: collective effects of exposure and behavioral characteristics without a priori age and parental myopia covariates; ^b^Multivariable models further adjusted for age and parental myopia.

*3 boys with missing parental myopia status.

As a sensitivity analysis, we also analyzed risk factors for myopia in a model that treated behavioral characteristics as categorical (thus, equal sample sizes across groups). Similar to the above analysis, near work was a statistically significant risk factor for myopia (data not shown). Finally, no statistical interaction between group and behavioral characteristics on presence of myopia were observed.

### Factors associated with spherical equivalent refraction

Univariable analysis (Table 5) showed that spherical equivalent refraction differed across group, with the ultra-Orthodox group having more negative (more myopic) refraction relative to the religious (0.87D (0.37, 1.38)) and secular groups (1.06D (0.51, 1.62)). This finding remained statistically significant in the fully adjusted multivariable model (religious versus ultra-Orthodox: 0.93D (0.19, 1.67); secular versus ultra-Orthodox: 0.99D (0.1, 1.89). While near work was marginally significant in the univariable analyses, in multiple regression models, increased near work was associated with a more myopic refraction (− 0.1D (− 0.19, − 0.02)) and remained significant in the adjusted model (− 0.11D (− 0.19, − 0.03)). For time in school and age learned to read, univariable analyses showed that there was a statistically significant more myopic refraction with increased reported time in school and with a younger age learned to read, but these did not remain statistically significant in the multivariable and adjusted analyses.

**Table 5:** Associations of spherical equivalent refraction with exposure and behavioral characteristics of religious group, time outdoors, near work, age learned to read, and hours spent in school (n = 168).

Exposure and behavioral characteristics	Univariable analysis $\widehat{\beta } (95\% CI)$	P value	^a^Multivariable analysis $\widehat{\beta } (95\% CI)$	P value	^b^Adjusted analysis $\widehat{\beta } (95\% CI)$	P value
Group		P < 0.001		0.006		P = 0.049
Religious	0.87 (0.37, 1.38)	P = 0.001	1.24 (0.46, 2.02)	P = 0.003	0.93 (0.19, 1.67)	P = 0.015
Secular	1.06 (0.51, 1.62)	P < 0.001	1.52 (0.58, 2.46)	P = 0.002	0.99 (0.1, 1.89)	P = 0.031
Time outdoors	0.14 (− 0.26, 0.53)	P = 0.493	− 0.09 (− 0.48, 0.3)	P = 0.655	− 0.02 (− 0.38, 0.34)	P = 0.905
Near work overall	− 0.09 (− 0.17, 0)	P = 0.053	− 0.1 (− 0.19, − 0.02)	P = 0.018	− 0.11 (− 0.19, − 0.03)	P = 0.007
Early readers (< 6 years old)	− 0.62 (− 1.07, − 0.16)	P = 0.009	0.62 (− 0.14, 1.39)	P = 0.106	0.61 (− 0.09, 1.32)	P = 0.086
Time in school	− 0.31(− 0.5, − 0.12)	P = 0.002	− 0.17 (− 0.37, 0.03)	P = 0.086	− 0.17 (− 0.35, 0.02)	P = 0.082

Reference categories: Religious group: ultra-Orthodox; Older Readers (> 6 years).

Each model was implemented with numerical and categorical exposure forms included into the model to safeguard model assumptions (not presented).

$\widehat{\beta }$ Estimated beta coefficients from mixed linear regression model, *CI* confidence intervals.

^a^Multivariable Analysis: collective effects of exposure and behavioral characteristics without a priori age and parental myopia covariates; ^b^Multivariable models further adjusted for age and parental myopia.

*3 boys with missing parental myopia status.

As a sensitivity analysis, we also analyzed factors associated with a more myopic refraction, treating behavioral characteristics as categorical (thus, equal sample sizes across groups). Similar to the previous models, near work remained as a significant risk factor a more myopic spherical equivalent (data not shown).

## Discussion

The iREAD study takes advantage of the unique behaviors of different populations of Israeli children to examine relationships with myopia. Baseline data from this longitudinal study revealed that distinct behaviors between ultra-Orthodox, religious, and secular Jewish boys are associated with the higher prevalence of myopia and more myopic refraction in the ultra-Orthodox group. Using both subjective and objective measures, findings showed that Ultra-Orthodox boys learn to read at a younger age, spend more hours in school, and spend less time outdoors than religious and secular boys. Greater daily near work and time in school increased the odds of having myopia. Being ultra-Orthodox was associated with a more negative spherical equivalent.

The iREAD study enrolled boys from three different school systems: ultra-Orthodox, religious, and secular. Previous studies have shown that ultra-Orthodox boys have a higher prevalence of myopia than religious and secular boys, with speculation that the higher prevalence is associated with increased near work demands^19,20,22^. The educational systems and lifestyles are very different for ultra-Orthodox, religious, and secular groups. Ultra-Orthodox Jews in Israel have a unique educational system for boys that involves intensive sustained near-work activity beginning at the age of three. Ultra-Orthodox schools are single sex, with the boys’ schools focusing mainly on intensive reading of religious texts, generally with small font. The number of study hours in ultra-Orthodox boys’ schools is greater than religious and secular schools (or ultra-Orthodox girls’ schools)^25,30^. Through studying these unique populations in Israel, we have extended findings from previous studies to a younger population and added objective measures of physical activity and light exposure to confirm that there are significant differences in time spent outdoors, but not activity, when measured continuously over 24 h and for several days.

The iREAD study successfully enrolled 168 boys who were balanced for age and season. As expected, the ultra-Orthodox group exhibited a higher prevalence of myopia than the secular boys and significantly more myopic refraction. Additionally, the ultra-Orthodox boys tended to have a longer axial length (P = 0.051), but this did not meet the criteria for statistical significance. It is possible that as the boys get older, prevalence of myopia in this population will increase and axial length differences may emerge as significant as this longitudinal study continues. Behaviors, as well as refraction, have been shown to be associated with age^31,32^. Additionally, ambient temperature and hours of daylight vary significantly with season (i.e. time of year) and impact children’s behavior^33^. By balancing for age and season, it is likely that the differences observed between children across groups was not due to age- or season-difference, and rather, reflect different cultural and educational styles.

While the sample size was relatively similar across groups, many ultra-Orthodox parents and some religious parents did not allow their children to wear the Actiwatch on Shabbat for religious reasons. This resulted in a smaller cohort for Shabbat. Ultra-Orthodox and religious Jews have traditions on Shabbat that are significantly different from secular Jews. Shabbat observance includes special prayer services in the synagogue, family time, and religious studies^34^. Shabbat-observant Jews refrain from specific activities, such as writing, going to work, riding in a car, and using electric appliances and devices^35^. Secular Jews typically do not refrain from these activities, but are on break from school and work during Shabbat.

Questionnaire data revealed that ultra-Orthodox boys spent more hours in school than the other groups and learned to read at a younger age, similar to findings reported in a previous study^21^. Interestingly, while not in school, the three groups exhibited a similar amount of near work; however, the type of near work was different between groups. Ultra-Orthodox boys tended to engage in near work that utilized traditional printed material, whereas religious and secular boys engaged in significantly more electronic device use than ultra-Orthodox boys. In terms of overall use of screens (hand-held devices, computers, TV/video games) secular boys spent significantly more time using screens than religious and ultra-Orthodox boys, and religious boys spent significantly more time using screens than ultra-Orthodox boys, aside from Shabbat when religious and ultra-Orthodox are similar. The literature reports conflicting findings regarding the role of screen time on myopia. There has been increased use of smart phones, tablets, and computers in recent years, and it has been suggested that these electronic devices exacerbate myopia^36^. While some studies show an association between screen time and myopia^37,38^, other studies find no link^39^. A recent meta-analysis reported that smart devices “might be associated with an increased risk of myopia,” but concluded that more objective measures of screen time and myopia-related outcomes that investigate smart device exposure as an independent risk factor are required ^40^. In the case of the ultra-Orthodox population studied here, it is extremely unlikely that the use of electronic devices is driving the high prevalence of myopia, given that this cohort has a significantly higher prevalence of myopia than other groups, with significantly less electronic device use.

Light exposure and time outdoors were assessed objectively using a wrist-worn light sensor. Overall, the ultra-Orthodox boys had significantly less light exposure and spent less time outdoors than the religious and secular boys. Ultra-Orthodox boys also spent significantly less time outdoors during the weekdays (Sunday-Friday) than the other groups, although this difference was not observed on Shabbat (Saturday). Given the longer school days reported by the parents for ultra-Orthodox boys it is not unexpected that they might experience less light exposure during the week. However, in our previous pilot study^21^, no statistically significant differences were found between the three groups for time outdoors. The boys in the current study are younger than the previous study, which may account for the difference. Furthermore, the previous study only included 36 children, while the current study has 168 children. The season that the pilot was conducted may also have impacted the results: the boys in that study wore the watches when school was in session from June 2019 to March 2020, whereas in the current study, they wore the watches from Nov. 2021 to June 2022. The first study included the months of September and October, which have long days, lots of sunlight and no rain, while the second did not.

When testing light exposure and activity across the 24-h weekday, ultra-Orthodox boys demonstrated less light exposure and physical activity during the morning while at school than secular or religious boys. It may be possible that ultra-Orthodox children do not play outside as much during morning recess in comparison to their religious and secular peers. While time outdoors did not emerge as a significant risk factor for myopia in this study findings show that behaviors are different during the school day. This suggests that younger ultra-Orthodox boys are at risk for even less light exposure, since they start school at age three. Given that early exposures may influence eye growth and myopia, it is necessary to examine the differences in light exposure during the school day for younger, preschool-age ultra-Orthodox, religious, and secular boys in future studies. If indeed light exposure during the school day contributes to the development of myopia, this is a risk factor that can be modified by active recess interventions^41^.

Increased hours in school and increased near work at home both increased the odds of developing myopia, while time outdoors did not. Furthermore, being ultra-Orthodox was associated with a more negative spherical equivalent refraction in comparison to being religious and secular, with nearly a 1 diopter difference between the ultra-Orthodox group and the other groups. The only behavioral factor that was associated with a more negative spherical equivalent was near work, with approximately 0.1 diopter difference for children that engaged in more near work. This model suggests that being ultra-Orthodox has a greater association with spherical equivalent refraction over and beyond behavioral characteristics, age and parental myopia. Taken together, the results suggest that the combination of group, behaviors, age, and parental myopia yield incongruent effects across the two outcomes of interest (the presence of myopia and spherical equivalent refraction). Increased near work and hours in school, but not group, increased the odds ratio of having myopia. On the other hand, being ultra-Orthodox was highly associated with a more negative spherical equivalent, with a smaller contribution of increased near work. The participants in this study were young children (ages 6–10 years) and it is possible that some will develop myopia in the future. Indeed, adolescent ultra-Orthodox boys have been reported to have both a higher prevalence of myopia and more negative spherical equivalent refraction than what was observed in the younger population included in the current study^19^.

Despite the ultra-Orthodox children spending less time outdoors, it did not emerge as a risk factor for myopia in this cohort, nor was it associated with more myopic spherical equivalent refraction. The average for all children was 1.2 h outdoors per day, significantly less than the 2 h daily recommended as an effective intervention for myopia^42^. Note that this study measured behaviors during the school year and not during vacation or summer breaks. It may be that the ultra-Orthodox children have less light exposure during school breaks. Indeed, ultra-Orthodox schools in Israel typically have 50 more school days than religious or secular schools. For example, the religious and secular schools have summer break for the entire months of July and August. In contrast, ultra-Orthodox schools only have three weeks of summer break from the 9th of the Hebrew month of Av until the 1st of the Hebrew month of Elul (typically in July or August). Alternatively, it may be that the ultra-Orthodox boys get less light exposure at a younger age than the children in this study, since they start formal school at age three. As an additional/ancillary component of the iREAD study is concurrently collecting exposure data on a smaller cohort of children during vacation months, hopefully to further explain light exposure differences in ultra-Orthodox boys.

Limitations of this study include the following. A questionnaire was used to assess near work and electronic device use. Questionnaires are subjective and rely on parental observation and recall. Some parents reported unrealistic observations regarding certain activities (for example, 16 h of near work a day, when not in school). Nevertheless, the same recall bias would apply to all the children, making it possible to compare between the groups. Another limitation was that the refractive status of the children's parents was not assessed by a clinical examination, but by self-reported survey. However, this survey has been shown to have reasonable sensitivity and specificity for self-report determining whether individuals ages 14–85 years are myopic^43^. It should be noted that the questionnaire asked parents to assess behaviors when the children were at home. Thus, aside from quantifying the number of hours children were in school, specific behaviors do not reflect visual activity during school time. This is a limitation for assessment of the time the children spent reading and writing, since they typically engage in extended time in near work during school. This limitation may have been addressed by simply asking parents how many hours per day the children spent in school. However, the questionnaire did not address the specific amount of time children were engaged in near work in school and parents may not have known. Additionally, children in Israel typically do not engage in electronic device use during school. Furthermore, we did not measure the religious orientation of the students, only the school system that they attended. However, the school systems in Israel are typically highly homogeneous and this definition of religious group has been used in previous studies on the prevalence of myopia and school systems in Israel^19,20,22^.

An additional limitation of the study is the sample size, which may have limited sensitivity to detect some effects, especially in the models with correlated variables. As in all research, confidence in the importance/unimportance of specific variables increases as findings are replicated in future research. However, this dataset is rich, with more than 1000 data points collected on each boy, is observational in nature, with no null hypothesis to prove or disprove, and is part of an ongoing longitudinal study, upon which primary analyses will focus on longitudinal changes.

In summary, this study reports the baseline characteristics and behaviors of boys enrolled in the Israel Refraction, Environment, and Devices (iREAD) Study.

Findings shows that ultra-Orthodox boys ages 6–10 years are more myopic than their religious and secular peers. Ultra-Orthodox boys spent less time outdoors and had less light exposure, primarily during the morning while in school. In addition, ultra-Orthodox boys spent more hours in school per day and learned to read at a younger age. Being ultra-Orthodox was associated with a more myopic spherical equivalent than being secular or religious. Additionally, risk factors for having myopia included increased time in school per day and increased near work at home, while time outdoors did not emerge as a significant risk factor. Data collected over the next 18 months in this longitudinal study will assess the progression of myopia in these three groups of children to further identify risk factors for myopia.

## Material and methods

### Participants and protocol

The Israel Refraction, Environment, and Devices (iRead) Study is an 18-month longitudinal study to assess risk factors for myopia in boys enrolled in three school systems: (1) ultra-Orthodox, (2) religious, and (3) secular, during the school year and summer vacation. Healthy boys, ages 6–10 years old, with best corrected visual acuity of 6/9 or better in each eye, were recruited from the greater Jerusalem area of Israel via word of mouth and advertisements posted at the Hadassah Academic College clinic and on social media. Data were collected from Nov. 2021 to June 2022. Informed consent was obtained from all subjects and/or their legal guardian(s). The study was approved by the ethics committee of Hadassah College and followed the tenets of the Declaration of Helsinki.

Children who had an ocular disease or pathology, strabismus, abnormal vision as a result of ocular trauma or surgery, systemic diseases that affect refractive error, contraindications to the use of dilation drops, and history or current use of any myopia control treatment were excluded^21^. Children with hyperopic cycloplegic spherical equivalent (SE) ≥  + 2.50D were excluded because hyperopia is most likely a different developmental process than myopia^44,45^, and the goal was to study risk factors for the development of myopia. Children are generally born hyperopic; those that remain hyperopic fail to emmetropize in early childhood. On the other hand, myopia generally develops during school age years, once a child has already emmetropized^46^. Children with astigmatism > 3.00D in either eye were excluded, due to the high prevalence of keratoconus in Israel^47,48^. Children were not excluded for anisometropia as long as both eyes had best corrected visual acuity better than 6/9.

In Israel, it is known that parents send their children to the school system based on the religious sub-group with which they belong. Therefore, children were classified based on the educational system at which they studied: (1) ultra-Orthodox, (2) religious, or (3) secular. The recruitment strategy was to schedule 12 children a week (four from each group) with a similar age (± 6 months) to match for age, daylight hours, and weather. Mean daylight duration (from timeanddate.com), daily temperature and rainfall (from Israel Government Portal for National Meteorological Service^49^) were measured in order to assed potential differences between the three groups for the specific time period each child wore the Actiwatch^21^.

### Clinical examination

Children underwent a complete eye examination, including visual acuity and subjective refraction. Axial length was measured three times in both eyes (LenStar, Haag-Streit AG, Switzerland), and the average for each eye was calculated. Following biometry, both eyes were dilated with 1% cyclopentolate and 0.5% tropicamide, and fundus photos were taken and reviewed during the exam by a retina specialist. Cycloplegic refractive error and corneal power were measured in both eyes by autorefraction (VX130, Visionix Luneau, Chartres, France), at least half an hour after instillation of drops and after ensuring lack of pupillary response. Three measurements were recorded, and the spherical equivalent refraction was calculated for each eye. Children were classified as myopic (≤ − 0.50 D)^50^, hyperopic (≥ + 2.50 D) or emmetropic (> − 0.50 D to + 2.50) based on average cycloplegic spherical equivalent of both eyes. A prescription was provided when required.

### Subjective questionnaire

Parents completed a visual activity questionnaire, adapted from the University of Houston Near work, Environment, Activity, and Refraction (UH NEAR) Survey and translated to Hebrew (Appendix 1, http://links.lww.com/OPX/A503)^51^ which has been described extensively in a previous study^21^. If the parents did not return the questionnaire, the child was excluded from the study. Parents were asked to provide information regarding the child’s demographics, ocular history, education, and near work behaviors, such as electronic device use. The questionnaire also had questions to determine the refractive status (myopic or non-myopic) of the parents by asking whether the child’s biological mother and father wore glasses, and if so, whether the glasses were for near, distance, or both and the age they started to wear glasses^43^. Questions related to education and near work included age at which the child learned to read, number of hours spent in school per day, school performance, and time per day engaged in near work and using electronic devices (outside of school). To quantify different activities, parents were asked to estimate time spent in various activities for weekdays (while not at school) and for Shabbat. Weekdays included Sunday through Friday afternoon, when children were typically in school, and Shabbat included Friday evening through Saturday, when children were out of school. Shabbat begins about one hour before sunset on Friday and ends at sunset on Saturday.

### Objective behavioral measurements

An Actiwatch Spectrum Plus (Philips Respironics, Bend, OR)^21^ was dispensed for each child to wear continuously for 10–14 days, unless a school holiday fell in the middle of the wear time, in which case the watches were dispended for up to 29 days. The Actiwatch has been described in detail previously, and is has been used in both children and adults in various applications^21,32,33,52,53^. The Actiwatch was configured to average data over 15 s epochs. Children received oral instructions to wear the device continuously without removing it for sleep or bathing and to take care not to cover the device with shirt sleeves or coats.

Children were included in the final analysis if they provided Actiwatch data for at least 4 days and 4 nights that met the inclusion criteria. Daytime data from the Actiwatch were included when the child wore the device for the entire day. Some children did not comply with the instructions not to remove or cover the Actiwatch. As a result, days were also excluded if the child removed the watch for more than 90 min, or if the light exposure dropped to zero for 60 min or more during daylight hours. Holidays and days when children were not in school (for example, due to illness) were excluded. Nighttime data were included when the watch was worn from bedtime to wake time.

Actiwatch data were downloaded for each child and the following parameters were assessed: minutes the device was “off wrist,” activity in counts per 15 s, white light (lux), and interval status (active or sleep status). A day was defined from 12:00:00 to 23:59:45. Bedtime was defined as the time when the interval status changed from active to non-active. Wake time was defined as when the interval status changed from non-active to active. Time spent outdoors during daylight hours was defined as minutes per day exposed to > 1000 lx. This cut-off point is based on the manufacturer’s recommendations, on a validation study in children^54^, and from previous studies of light exposure and vision in children which used a > 1000 lx value to indicate outdoors^21,32,33,53,55–58^. Total activity was calculated as mean daily counts per 15 s. Moderate-to-vigorous physical activity was defined as the number of minutes per day that each subject spent performing activity greater than 1048 counts per minutes, based on a validation study of the Actiwatch and different types of physical activity in children^59^ and previous research in children^32^.

### Data analysis

Mean daily behaviors from both the questionnaire and Actiwatch data were analyzed separately for weekdays (bedtime on Saturday night to bedtime on Friday night) and for Shabbat (bedtime on Friday evening to bedtime on Saturday evening). Overall behavior for the entire week, i.e. “overall,” was calculated using Eq. (1)^21^.

1 $${\text{Mean daily behavior overall }} = \, \left( {\left( {{6 }*{\text{ mean of weekdays}}} \right) \, + \, \left( {\text{mean of Shabbat}} \right)} \right)/{7}.$$

Data were downloaded from each watch into Excel using the Actiware Software (Actiware 6.1.1.3, Philips, Respironics, Inc.). The software provides illumination in lux and physical activity in counts per 15 s epoch. Means and standard deviations are reported by condition.

Mean daily light exposure (lux), hours per day spent outdoors (> 1000 lx), and mean daily activity (counts per 15 s epochs (CP15)) were calculated for each subject for weekdays, for Shabbat, overall as well as across 24 h a day for weekdays, Shabbat, and overall for the three groups of children. Physical activity classifications of sedentary (< 80 CP15), light (80 to < 262 CP15), moderate (262 to < 406) and vigorous (> 406 CP15), as reported in the literature^59^, were applied to the observed data. For the purpose of this study, moderate and vigorous physical activity were combined. Frequency of physical activity conditions and classifications were computed.

Activities on the survey were categorized as near (< 40 cm), intermediate (40 to 100 cm), and far work (> 100 cm)^21,51^.

### Statistical analysis

Child and family demographics, clinical characteristics, survey items, and Actiwatch metrics were summarized by means with standard deviations, medians with interquartile ranges, minimum/maximum values, or frequencies/percentages depending on whether the variable was categorical or numerical (ordinal versus continuous). Histograms and quantile–quantile plots were examined, and skewed variables were log-transformed where relevant and collapsed into categorical variables in some cases. Overall and group summary statistics were computed. Group comparisons among family level variables were analyzed under the assumption of independent observations. Thus, family level group effects were assessed using one-way ANOVA, Kruskal Wallis, and Fisher’s exact tests, as appropriate. To assess child level measurements, children nested within families (i.e. participants in the study that are siblings) were taken into account using generalized estimating equations and generalized mixed effects models with random effect for family and the main effect of group was tested. In cases of categorical outcomes, the logit link function was specified. In cases where transformations were not applicable and distributional methods could not be performed on the raw observed measures, statistical analysis was performed on the ranked values. In the presence of a statistically significant main effect of group, post hoc comparisons were made. Type I error was safeguarded against at the metric level when assessing Type III effects as well as during post hoc testing. Hypothesis testing was assessed at the 0.05 level. A similar approach was used to assess Shabbat versus weekday behavioral differences, measured via the Actiwatch, across groups. Main effects included within subject variable week component (weekday versus Shabbat), group and the interaction between week component and group. To assess the differential effects of age, children were classified as younger (5–8 years) or older (≥ 9 years), and group by age Actiwatch metrics were evaluated. A similar repeated measure mixed models analysis was conducted to assess the differential effects of group on hour by hour aggregate measures of activity and white light exposure for weekdays and Shabbat, separately, via a group by hour interaction followed by post hoc testing.

Given prevalence estimates, we hypothesized that the ultra-Orthodox group was at increased odds of myopia. To assess the relationship between group and account for the contribution of behavioral characteristics and their relationship to myopia, we used generalized linear mixed models with a logit link function and random effect for family to calculate odds ratios (OR) and their 95% confidence interval (CI). Thus, multilevel multiple logistic regression was used to analyze the relationship of group, behavioral risk factors, and other variables with binary outcome of refractive group, myopic versus non-myopic. Given the concerns of the analytical form of some of the numerical variables (e.g. overall self-reported near work, self-reported hours in school, and age learned to read) in interpretation of model results, we examined relationships with myopia, treating exposures as continuous variables as well as categorical (binary and tertile where relevant). Age first learned to read was categorized at school entry (< 6 years or > 6 years). Time outdoors and near work were categorized using tertiles and hours in school was categorized at the median level. This approach allowed the interpretation of the variable in its natural form as well as an approach that had equal representation of children across groups. A series of multivariable models were constructed (as laid out below). Further, the contribution of behavioral risk factors was assessed individually and collectively. Interactions between risk factors and group were assessed. Covariates age at entry into the study and genetic indicator number of myopic parents were specified a priori.

Univariable models were constructed to estimate the odds of myopia presence for group and individual behavioral risk factors. The next steps were conducted in a phased approach. First, individual and collective effects of group and behavioral risk factors were assessed in multivariable models without covariates; second, models from phase 1 that included age and myopia of parents were included in fully adjusted models. Interactions between group and behavioral risk factor was tested. Variance inflation factors were used to examine signs of multicollinearity. Results of univariable and multivariable models are presented with estimated odds ratios (OR) and 95% confidence intervals (CI).

In a similar way, multiple linear regression via mixed effects models were used to analyze the relationship of religious group, behavioral risk factors, and other variables with the degree of myopia with continuous outcomes axial length and spherical equivalent refraction. Results of univariable and multivariable models of spherical equivalent refraction are presented with estimated beta coefficients and 95% CI. Statistical analyses were conducted using SAS Version 9.4. All statistical analyses were performed based on a complete-case analysis approach.

### Ethical approval

All procedures performed in studies involving human participants were in accordance with the ethical standards of the institutional and/or national research committee (Hadassah Academic College Ethics Committee) and with the 1964 Helsinki declaration and its later amendments or comparable ethical standards.

### Informed consent

Informed consent was obtained from all individual participants included in the study.

## Data Availability

The datasets used and/or analyzed during the current study available from the corresponding author on reasonable request.
